# Resume of Recent Discoveries and Improvements in Medicine

**Published:** 1871-09

**Authors:** Wm. Abram Love

**Affiliations:** Atlanta, Georgia


### RESUME OF RECENT DISCOVERIES AND IMPROVEMENTS IN MEDICINE.

By Wm. Abram Love, M.D., Atlanta, Georgia.

### TOXICAL EFFECTS OF CHLORAL HYDRATE WHEN PERSISTENTLY USED AS A HYPNOTIC, AND FATAL RESULTS OF LARGE DOSES.

Dr. N. R. Smith, of Baltimore, reports ("Boston Medical and Surgical Journal") two cases in which the long continued use of chloral hydrate, as a hypnotic, had been followed by "a singular affection of both hands, attended with desquamation of the cuticle and superficial ulceration, especially about the borders of the nails, * * attended with pain and much morbid sensibility to touch; * * also associated with some acceleration of pulse and general malaise."

The first case (M., æt. seventy,) was followed—after cure of local affections—by acute bronchitis, embarrassed respiration, hoarse mucous râle, enfeebled heart's action, and, ultimately, death.

The second case (F., æt. twenty-two,) had been using chloral nightly for a month; suffered with precisely same local affections about the fingers, followed by extreme illness, universal anasarca, feeble heart's action; pulse one hundred and forty; extremely weak; respiration much embarrassed; recumbent posture impossible; urine albuminous. Under the use of stimulants, diuretics, and digitalis, she made good her recovery.

Dr. Smith also alludes to two other cases in which the same affection of the fingers resulted from the use of chloral.

About the time this paper was presented to the profession of America, Dr. Hanfield Jones, of London, (if our memory is correct), called the attention of the profession on the other side of the water to the advantages of the same plan of treatment.

In one case in our own practice (in 1859), we succeeded, by the *alternate* use of the warm cerebro-spinal baths and cold douches (momentary), together with artificial respiration, in establishing respiration after the lapse of one hour and twenty minutes from birth. In our opinion, many children passed as *still-born* might, we think, by persistence in this plan, be resuscitated.

He reports two deaths occurring in Baltimore, within ten days, "manifestly from the toxœmia caused by an over-dose of chloral."

In one case, the patient persisted in the use of the hydrate after his physician had ceased attendance, taking it in half-drachm doses; the other case "was attended by a Homeopathic physician,"—the dose given not larger than has been recommended as safe. In both these cases, the death was sudden and unexpected. He also alludes to another case, occurring in Washington, where death followed the administration of a drachm and a half, by enemata, to allay pain resulting from a surgical operation,—death attributed to the chloral.

Dr. Smith says, in conclusion:

"These cases are, it appears to me, amply sufficient to establish the toxical effects of this powerful agent. It is probable that its poisonous effects are exerted in two ways,—

"1. When given in a large dose, and especially where the system may have been charged with it by its previous administration, it at once overwhelms the powers of life, and causes immediate death.

"Upon what organ or organs does it exert its deadly effects? It must be either upon the heart or brain,—perhaps on both.

"It is believed that chloral, entering into the blood, develops chloroform in that fluid—the amount developed being determined, not merely by the quantity taken, but by the condition of that fluid. Chloroform, we know, when respired, exerts its influence upon both brain and heart. In the numerous cases in which it has caused death, this result has been produced by its interrupting the circulation.

"2. It appears, when given in small doses, and continuously for some time, to induce a form of toxœmia similar to that caused by the continued administration of ergot. Its effects on the fingers of both hands in the two cases related above, would justify such a belief. It is well known that animals fed on spurred rye suffer gangrene of the extremities."

Dr. Smith regards the free and repeated use of *chloroform* as injurious, producing chronic poisoning of the blood, and, as secondary effects (following operations), secondary hemorrhage, pyœmia, erysipelas, and hospital gangrene.

QUINIA SULPHATIS AS AN ALIMENT SUBSTITUTING TAURINE OR TAUROCHOLIC ACID, AND ITS ACTION IN GIVING TONICITY TO CELL-DEVELOPMENT.

Dr. A. S. v. Mansfielde, of Chicago, Illinois, ("Medical and Surgical Reporter,") takes the position that sulphate of quinia, in its action, substitutes, in the economy, the taurine or taurocholalic acid generated by the liver; that the want of this aliment, when it is not secreted, or not reabsorbed after entering the duodenum, leads to loss of proper tonicity in the cell-formations, and, as a sequence, to the breaking down or arrest of normal developments of the cells of most, or all, of the tissues. He holds that this substitute "restores the normal condition of organs whose functions have been perverted through the absence of taurine." In all such cases, he says, "the same pathological conditions and the same symptoms spring up to sight in all disorders that take their origin (either supposed or proven) from a suspended secretion or excretion of bile.

"We find in all these diseases exudates into the cavities, and a tendency to general dropsy, not dependent upon heart or kidney disease. In all of them, exists a disposition to fluxes of different compositions and localities; the epithelial cells collapse, and are destroyed, (by breaking down of the cell-walls), leaving their former position denuded. In all of them, we find a lessened amount of fibrin in the blood, a decrease of animal heat, a general debility, a marked dryness of the muscle tissue, and a collapse of this and all other soft cellular structures. In all of them, life is ended by asthesia, etc. * *

"*All of these conditions are the consequence of the suspension of one physiological act*,—THE NON-ABSORPTION OR NON-SECRETION OF TAURINE,—and are to be explained by one pathological condition; the collapse of the soft cellular tissues, and the prevention of the ofttimes dangerous manifestations of such a state of affairs, lies in the hands of the physician, by substituting for the organic aliment the artificial one, until the former may again be furnished in amount equivalent to the demand, and such a valuable article is *quinia sulphatis*."

LACTO-PHOSPHATE OF LIME.

Prof. R. W. McCready ("New York Medical Journal") strongly recommends the use of this remedy in cases of depraved or imperfect nutrition. He cites the experiments of Milne-Edwards and M. Durart, instituted to show its influence in hastening and increasing the production of callus after fractures, its benefits in rachitis, and its advantages in diarrhea and indigestion after other plans of treatment had failed,—attributes these benefits to its influence in the process of cell-formations and its power of increasing the plastic materials in the blood. Has found it useful—

"1. In cases of defective nutrition, with or without diarrhea, but without any acute disease of the alimentary canal—particularly when these conditions have occurred in prematurely-weaned children.

"2. In rachitis.

"3. In atonic dyspepsia.

"In most of these cases, not only were the digestive power and nutrition of the patient greatly improved, but the appetite for food was augmented, sometimes to an extraordinary degree.

The preparation used by Dr. McCready is obtained by preparing the phosphate of lime as in U. S. P.—action of muriatic acid on bone earth, and precipitating phosphate with ammonia.

In one ounce of concentrated lactic acid, dissolve freshly-precipitated phosphate to saturation; to this clear solution, add six and a half ounces of water, an ounce and a half of orange flower water, and twelve ounces of sugar. Each ounce thus prepared represents fifteen to twenty grains of the phosphate, varying according to the strength of the lactic acid. Dose—for young child, one to two drachms, for an adult, table-spoonful, three or four times a day; taste pleasantly acid—not apt to disagree with even a delicate stomach, and may be taken up by lacteals without undergoing decomposition by the gastro-intestinal juices.

### MOISTURE IN PROMOTING THE SPREAD OF ERYSIPELAS, PYÆMIA, GANGRENE, AND OTHER KINDRED DISEASES.

Assuming that pus-cells of a specific character are concerned in propagating this class of diseases, Dr. Day, of Geelong, has demonstrated the influence of moisture and moist atmosphere in promoting their spread. He says (before Medical Society of Victoria—Melb. Arg.—"Medical Times and Gazette):

"In 1868, I had the good fortune to discover a very delicate test for pus, and have since been in the almost daily habit of applying it, in conjunction with other tests, as aids to diagnosis. I have found that *healthy pus, when dried*, becomes *chemically inactive*, although *when moistened* with water, *it again resumes its chemical activity.* Also, that *strumous pus* possesses much *less chemical activity* than *pus* derived from *healthy persons;* and that the *pus* from persons suffering *from diseases allied to erysipelas*, possesses *unusual activity*, which it is *capable of retaining for years.* On this piece of glass [he demonstrates] is some pus taken from a large carbuncle on the neck of an elderly gentleman, two years and three months ago. He was suffering from symptoms of blood-poisoning at the time. This pus, although it has been freely exposed to the air during the whole time, and sometimes to great heat, still retains its power of acting chemically on the pus-test; and it does so even when dry, thus showing that it possesses greater chemical activity than ordinary pus.

"In the explanation I have attempted regarding the influence of moist and dry air over the propagation of erysipelas and its allied diseases, I have assumed that *when the chemical activity of pus is suspended, its power to act as a poison on the system is also suspended.* I have found that *carbolic acid*

*possesses the property of entirely and permanently destroying the chemical activity of pus, whether derived from healthy or unhealthy persons.*

"'On this paper [he demonstrates again] is some pus which had been moistened with water to give it chemical activity. A few drops of watery solution of carbolic acid were then poured over it, and after a lapse of a quarter of an hour, the pus-text was applied with a perfectly negative result.'

"Dr. Day *prepares* his *test-fluid* by *exposing a saturated alcoholic solution of guaiacum to the air until it has absorbed a sufficient quantity of oxygen to give it the property of turning green when placed in contact with iodide of potassium.* On moistening the most minute quantity of pus with water, and pouring a drop or two of the test fluid over it, a clear blue color is produced."

Dr. Day, but a few years since, gave to the profession his most excellent test for *blood*, which has been found reliable, and now he adds another for *pus*. This, with the results of experiments with it, will certainly serve to throw more light on the subject of the influence of moisture and moist atmosphere in the spread of some forms of disease, as well as to aid in their prophylaxis, by understanding better their cause. A better knowledge of their etiology and pathology will lead to a more successful plan of treatment.

We cannot, at all times, control the hygrometric condition of the atmosphere, or prevent those changes which every observing army or hospital surgeon has seen result in the rapid spread of gangrene, erysipelas, and kindred affections; still, with the antidotal or neutralizing action of carbolic acid, we may protect our patients against the poison; and it is not impossible that a system of atomizing may ultimately lead to the neutralization of the chemically-active specific pus in contaminated wards, and abolish this form of disease. Prof. Sextorph, of Copenhagen, ("Lancet"—Braithwait's Retrospect), reports, even now, the abolition of pyæmia from his ward, for twelve months, by the antiseptic plan of treatment pursued by Prof. Liston, of Glasgow,—the free use of carbolic acid and carbolic solutions, and the utmost caution in dressing wounds, to prevent putrefaction and profuse suppuration.

These results are certainly most encouraging, and will evidently lead to great results in after-treatment by saving to the system of patients vital force, as well as nutritive material lost in the suppurative process.

### SKIN GRAFTING.

Transplantation of skin by Reverdin's method has been practiced by several physicians and surgeons, both foreign and American; and the reports from most of those who have experimented in this line favor the operation as materially aiding in bridging over old and extensively-ulcerated surfaces. The stimulus of these new centers or little islands, planted here and there in the ocean of granules constituting the extensive ulceration, not only give new vigor to the immediate surrounding cell-forms, but stimulates the more perfect development of the shore-cells at the nearest border-points. Nor does the influence cease here. The cell-germs along the line from island to nearest shore-point strengthen and mature; but an isthmus of cuticular membranes is rapidly built up, bridging the ocean from shore to island, when the line of force of stimulation changes from bridge to bridge, and the whole surface is covered with cuticular tissue, and the ulcer is healed.

Czerney has succeeded ("Lancet") in grafting the epithelium of an extirpated nasal polypy on the granulating surface of a wound, still preserving its ciliated character.

The human skin has been successfully planted on the dog (Russia), and that of the lizard on the frog (Vienna). Skin from healthy amputated limbs has been planted, with good results (Bristol), in the human subject.

The size of the grafts used have varied, with different operators, from a mere speck, embodying cells of the basement membrane, to the size of a grain of rice and (as Czerney's) to the size of a cent piece.

A goodly number of satisfactory results are reported (exchanges) from different sections, in cases of extensive ulcerations following burns, etc., where other plans of treatment, persisted in for years, had failed of the desired end.

### WARM CEREBRO-SPINAL BATH IN CONGENITAL APNŒA.

Dr. E. D. McDaniel, of Camden, Alabama, (Transactions American Medical Associations, 69), strongly advocates the use of the *warm occipito spinal or cerebro-spinal bath in cases of apnœa of new-born infants*, where proper respiration can not be induced or established by the ordinary means. He advises the application of the warm water "to the posterior part of the body, leaving the anterior out." * * * "It promotes nerve-action, and thereby respiration, by improving the circulation in the cerebellum, medulla-oblongata, and medulla-spinalis." From this, and a resort to artificial respiration, he reports gratifying results, *after many years of experience in the practice.* He refers to cases where this plan had resulted in the establishment of respiration and circulation ten to twenty minutes after delivery: one case "in which the first inspiration took place thirty minutes after birth; another, ninety minutes; another, two and a half hours; and another, four hours."

